# Prevalence of abnormal findings when adopting new national and international Global Lung Function Initiative reference values for spirometry in the Finnish general population

**DOI:** 10.3402/ecrj.v3.30658

**Published:** 2016-09-06

**Authors:** Annette Kainu, Ari Lindqvist, Anssi R. A. Sovijärvi

**Affiliations:** 1 HUCH Heart and Lung Center, Peijas Hospital, University of Helsinki and Helsinki University Hospital, Helsinki, Finland; 2 Research Unit of Pulmonary Diseases, University of Helsinki and Helsinki University Hospital, Helsinki, Finland; 3 Department of Clinical Physiology and Nuclear Medicine, University of Helsinki and Helsinki University Hospital, Helsinki, Finland

**Keywords:** spirometry, reference values, GLI2012, obstruction, restriction, severity grading

## Abstract

**Background:**

New Finnish (Kainu2015) and international Global Lung Function Initiative (GLI2012) reference values for spirometry were recently published. The aim of this study is to compare the interpretative consequences of adopting these new reference values with older, currently used Finnish reference values (Viljanen1982) in the general population of native Finns.

**Methods:**

Two Finnish general population samples including 1,328 adults (45% males) aged 21–74 years were evaluated. *Airway obstruction* was defined as a reduced ratio of forced expiratory volume in one second (FEV1)/forced vital capacity (FVC), *possible restrictive pattern* as reduced FVC, and *decreased ventilatory capacity* as reduced FEV1 below their respective 2.5th percentiles. The severity gradings of reduced lung function were also compared.

**Results:**

Using the Kainu2015 reference values, the prevalence of airway obstruction in the population was 5.6%; using GLI2012 it was 4.0% and with Viljanen1982 it was 13.0%. Possible restrictive pattern was found in 4.2% using the Kainu2015 values, in 2.0% with GLI2012, and 7.9% with the Viljanen1982 values. The prevalence of decreased ventilatory capacity was 6.8, 4.0, and 13.3% with the Kainu2015, GLI2012 and Viljanen1982 values, respectively.

**Conclusions:**

The application of the GLI2012 reference values underestimates the prevalence of abnormal spirometric findings in native Finns. The adoption of the Kainu2015 reference values reduces the prevalences of airways obstruction, decreased ventilatory capacity, and restrictive impairment by approximately 50%. Changing from the 2.5th percentile, the previously used lower limit of normal, to the 5th percentile recommended by the American Thoracic Society/European Respiratory Society will not increase the prevalence of abnormal findings in the implementation of spirometry reference values.

Spirometry is one of the most common clinical lung function tests. Changes in interpretation criteria will affect a large numbers of subjects and influence the diagnostic sensitivity of pulmonary diseases such as asthma and chronic obstructive pulmonary disease (COPD) (1, 2). Lung function is mostly dependent on sex, age, height, and ethnicity. Therefore, the selection of appropriate reference values is essential. Different reference values produce widely differing predictions for normality (3), a problem that has resulted in an effort to produce global reference equations. The Global Lung Function Inititiative (GLI) produced the first global reference equations in 2012 (GLI2012) by collating reference data from different research groups and countries to produce all-age reference values with continuous predictions from childhood to old age (4, 5). Data from different locations have been grouped according to mathematical similarity with less focus on preconceived ethnic similarity. Thus the largest group, ‘Caucasian’, is a very diverse category, encompassing geographically varying locations and ethnic backgrounds from North and South America to Europe, Australia, Asia, and Northern Africa (4).

There are yet very limited comparative data on the practical implications of using the GLI2012 reference values in different general populations. In Tunisia, Northern Africa, the Caucasian prediction equations have been shown to overestimate lung volumes, with mean z-scores for healthy non-smokers of forced vital capacity (FVC) of −0.62 (SD 0.86) and of forced expiratory volume in one second (FEV1) of −0.55 (SD 0.87) (6). In a primary care cohort from the Netherlands, the grading of reduced lung function in COPD was milder with the GLI2012 reference values compared to the old European Coal and Steel Community (ECSC) reference values, also due to the same prediction difference (7). In Northern Sweden, the GLI2012 reference values have been found to underestimate lung volumes with an increasing age trend and also to overestimate the prevalence of obstruction in females (8). In patient samples from hospital laboratory spirometry databases in Australia and Poland, it has been shown that the GLI2012 reference produces significantly higher predictions for lung volumes compared to the previously used ECSC reference values but a smaller difference when comparing with the National Health and Nutrition Examination Survey III reference data set in the United States (9, 10).

In some countries, different reference values are used in different parts of the country, which can make interpretation difficult when patients move from one area to another. In Finland, the choice of reference values for native Finns has been agreed upon and a uniform interpretation of test results has been in practice for over 30 years (11). Currently, two sets of reference values are used systematically for clinical interpretation of spirometry for native Finns: one for adults and the other for children (12, 13). The Viljanen1982 reference values for adults were formed with an occupational health cohort in late 1970s using the equipment and methodology of that era. It has become necessary to transition from these established, but outdated, reference values to new values recorded with modern transducers and that include elderly subjects. The implications of adopting the recently published new reference values for native Finns (Kainu2015) are not yet known (14).

New Finnish reference values have been developed using similar modelling with the recently published GLI2012 reference values (4, 14). In the healthy reference population of native Finns, the GLI2012 reference equations underestimated FVC by 6.2% in males and 5.1% in females with an age-dependent increasing trend (14). In Brazil, the GLI2012 predictions were similarly found to underestimate lung volumes in the reference values population of white adults, especially in males, and in older adults (15). In Japan, the secular trends in the relationship between sitting and standing heights, that is, the Cormic index, have been suggested to explain up to 50% of the age-specific variation in mean FEV1 and FVC z-scores among healthy non-smokers and to justify the continued need for national reference values (16, 17). However, it is important to evaluate reference values in random population samples to assess the validity of the models and the implications of changing reference values.

The aim of this study was to compare the differences in interpretation of spirometry results when applying the new Finnish reference values by Kainu (14), the international GLI2012 reference values (4), and the previously used national reference equations by Viljanen (12) in a random population sample of native Finns. The categories of spirometric findings studied are *airway obstruction*, *possible restrictive pattern*, and *decreased ventilatory capacity*, that is, decreased FEV1. The gradings of reduced FEV1 were also compared.

## Methods

We used the original population data from the FinEsS study from two centres, Helsinki and Kemi (14, 18, 19). Data from 633 subjects in Helsinki and 695 in Kemi with an acceptable baseline spirometry were included in the present study. The original studies were accepted by the Helsinki University Central Hospital and Länsi-Pohja Hospital boards of ethics, and all subjects gave informed consent. Only data from native Finns and spirometry values before an eventual bronchodilator test were used. The spirometry procedure has been published in detail elsewhere (14). The measurements were undertaken using the American Thoracic Society (ATS) 1994 criteria (20). The methods are described further in the Supplementary file. The spirometry variables evaluated were FEV1, FVC, and the FEV1/FVC ratio.

The ATS/European Respiratory Society (ERS) Task Force for spirometry (21) and the ATS 1994 criteria (20) define the *lower limit* as the 5th percentile of predicted values; this recommendation has been implemented in many national reference values. In Finland, it has been recommended to use the 2.5th percentile of the national reference values suggested by Viljanen et al. to define an abnormally reduced lung function in interpretation and a grading of the decrease based on standard deviations (12, 22). In the new international GLI2012 reference values, the 2.5th percentile limit of z-score −1.96 was suggested for screening and case finding in random populations, whereas the 5th percentile limit of z-score −1.645 was suggested for clinical use to define abnormality when evaluating patients with known lung disease or symptomatic individuals (4). Quanjer et al. evaluated the clinical implications of changing reference values in a tertiary care unit in Australia and Poland and suggested the use of FEV1/FVC<LLN (lower limit of normal) and FEV1 z-score <−2 for airway obstruction and using z-score −1.645 to define the LLN (10, 23).

In this study, we used the 2.5th percentile limit to define LLN in all evaluated reference models and defined *airway obstruction* (or *airflow limitation*) as FEV1/FVC<LLN and a *possible restrictive pattern* in spirometry as FVC<LLN. *Decreased ventilatory capacity* was defined as FEV1<LLN. For z-scores, the 2.5% limit was rounded to −2 as proposed by Quanjer et al. (23). For comparison of practical impact, the use of the z-score limit of −1.645 is also evaluated. In assessment of differences between prediction models, a significant z-score difference of ±0.3 SD was used as proposed by Quanjer et al. (24).

For grading of reductions in lung function, the FEV1 relative to the reference standard was used following the ATS/ERS standard for interpretation of spirometry (21). By using the GLI2012 and Kainu2015 reference values, a uniform categorisation was implemented using values of the z-score between LLN and −2 for mild, −2 to −2.5 for moderate, −2.5 to −3 for moderately severe, −3 to −4 for severe, and below −4 for very severe reduction in FEV1 (23). For the Viljanen reference values, the *LLN* is defined as the 2.5th percentile corresponding to a z-score of −2. The published and currently used categorisation of reduced FEV1 has defined values above 80% (2.5th percentile LLN) as *normal* and between 65 and 79% as *mild reduction* (22). To create a comparable categorisation with the GLI2012 and Kainu2015 values, in this study, we defined values above 80% as *mild*. Further reductions in FEV1 were categorised based on established division on standard deviations of the original prediction model, with percent predicted limits of 79–65% considered moderate (2–3.5 SD), 64–45% (3.5–5.5 SD) moderately severe, 44–25% severe (5.5–7.5 SD), and below 25% (<7.5 SD) very severe reduction in FEV1 (22).

The predicted values, LLN, z-score, and percentiles of FEV1, FVC, and FEV1/FVC for each study participant according to the GLI2012 and Kainu2015 reference equations were calculated with the statistical program R (version 2.15.1, www.cran.r-project.org). For GLI2012 values, the macro provided by the GLI (www.ers-education.org/guidelines/global-lung-function-initiative/tools/r-macro.aspx) was used. All other statistical analyses were conducted using the statistical program SPSS (IBM SPSS Statistics for Macintosh, Version 22.0. Armonk, NY: IBM Corp.). Correspondingly, predicted values and percentiles according to the Viljanen1982 reference equations were calculated for all subjects, but since the model is valid only for adults 18–65 years, the range of extrapolated values are indicated in tables and graphs. The kappa statistic was used to evaluate the level of agreement between different categorisations between the evaluated reference standards (25). The chi-square test was used for categorical comparisons. The Student's *t*-test for paired samples was used to test statistical significance between continuous variables of the predicted values for FVC, FEV1, and FEV1/FVC with Viljanen1982, GLI2012, and Kainu2015. A *p*-value of 0.05 was chosen for statistical significance for all analyses.

## Results

The population sample consisted of 597 (45.0%) males and 731 (55.0%) females. The mean age was 48.8 (SD 13.2) years in males and 48.2 (SD 12.9) years in females with a range of 21–74 years in both genders. The mean BMI was 26.1 (SD 4.5) with a range of 16.9–53.3 kg/m^2^. The mean height was 176.5 (SD 6.6) cm and 163.0 (SD 6.1) cm for males and females, respectively. A summary of measured spirometric data and predicted values for the entire population sample stratified by sex are shown in Table 1.

**Table 1:** Summary of measured spirometric data and predicted values by Kainu2015 (14), GLI2012 (4), and Viljanen1982 (12) in a general population sample of native Finns*

	Measured data		Mean predicted value			Measured value% predicted mean (SD)		
			
	Mean (SD)	Range	Kainu2015	GLI2012	Viljanen1982	Kainu2015	GLI2012	Viljanen1982
FVC (L)								
Male	4.89 (0.95)	2.18–8.03	5.17§	4.90§	5.07§	94.1 (12.7)	99.7 (12.9)	96.2 (12.6)
Female	3.49 (0.63)	1.68–5.39	3.65§	3.48§	3.59§	95.6 (11.8)	100.5 (12.3)	97.7 (12.3)
FEV1 (L)								
Male	3.79 (0.86)	1.02–6.15	4.03§	3.89§	4.12§	93.6 (15.5)	97.1 (15.8)	91.5 (15.2)
Female	2.78 (0.56)	0.99–4.50	2.91§	2.82§	2.96§	95.6 (12.6)	98.9 (13.0)	94.3 (12.6)
FEV1/FVC (%)								
Male	77.2 (8.3)	34.8–98.4	77.9§	79.5§	81.3§	99.0 (10.0)	97.0 (9.9)	95.0 (10.0)
Female	79.6 (6.5)	44.7–98.6	79.4§	81.3§	82.4§	100.2 (7.5)	97.9 (7.4)	96.7 (7.3)

* Males, *n*=597; females, *n*=731

§ *p*-value for paired *t*-test between predicted values with Viljanen1982 and Kainu 2015, Viljanen1982 and GLI2012 and Kainu2015 and GLI2012<0.001. FEV1=forced expiratory volume in one second; FVC=forced vital capacity; GLI=Global Lung Function Initiative.

**Fig. 1:**
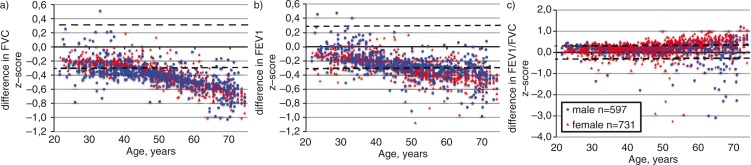
Difference between Kainu2015 (14) and GLI2012 (4) z-scores in a random general population sample of native Finns for (a) forced vital capacity (FVC), (b) forced expiratory volume in one second (FEV1), and (c) the FEV1/FVC ratio. Significant difference limits of ±0.3 SD as proposed by Quanjer et al. (24) are shown as dashed horizontal lines.

The difference in population distribution of z-scores of the Kainu2015 and GLI2012 reference values by age category is shown in Fig. 1. The GLI2012 produced lower predicted lung volumes than the Kainu2015 reference values and thus the FVC and FEV1 z-scores are slightly higher on average, especially in the older age groups. When evaluating the FEV1/FVC ratio, all subjects who showed normal values when assessed using the Kainu2015 reference value (z-score >−2) were also normal when assessed with the GLI2012 values. Compared to the currently used Viljanen1982 reference values, both the GLI2012 and Kainu2015 prediction models resulted in fewer subjects with values categorised as abnormal, as shown in Table 2.

**Table 2:** Prevalence of obstruction (FEV1/FVC<LLN) and possible restrictive pattern (FVC<LLN) with both 2.5th and 5th percentile limits for Kainu2015 (14) and GLI2012 (4) reference values and the established 2.5th percentile LLN for Viljanen1982 (12) stratified by sex and applicable age category

			Obstruction (FEV1/FVC<LLN)			Possible restrictive pattern (FVC<LLN)			Decreased ventilatory capacity (FEV1<LLN)		
					
Reference value	LLN	Age category	Male (%)	Female (%)	All (%)	Male (%)	Female (%)	All (%)	Male (%)	Female (%)	All (%)
Kainu2015	2.5%	All	7.4*	4.2*	5.6*	5.2*	3.4*	4.2*	8.0*	5.7*	6.8*
		21–65 years	5.9*§	4.1*§	4.9*§	4.3*§	2.9*§	3.5*§	6.6*§	5.0*§	5.7*§
	5.0%	All	9.7*	7.3*	8.4*	9.4*	7.1*	8.1*	12.6*	8.9*	10.5*
		21–65 years	8.2*	7.5*	7.8*	7.8*	5.6*	6.6*	10.5*	8.1*	9.2*
GLI2012	2.5%	All	6.0*	2.3*	4.0*	3.2*	1.0*	2.0*	5.5*	2.7*	4.0*
		21–65 years	5.3*†	2.1*†	3.5*†	2.7*†	0.8*†	1.6*†	4.5*†	2.7*†	3.5*†
	5.0%	All	9.0*	4.5*	6.6*	5.0*	3.4*	4.1*	9.0*	5.6*	7.2*
		21–65 years	7.8*	4.4*	5.9*	4.1*	3.1*	3.5*	7.4*	5.2*	6.2*
Viljanen1982	2.5%	21–65 years	11.5^§†^	10.2^§†^	10.8^§†^	7.6^§†^	6.3^§†^	6.9^§†^	13.9^§†^	9.2^§†^	11.2^§†^

* Kainu2015 versus GLI2012 *p*<0.001

§ Kainu2015 versus Viljanen1982 *p*<0.001

† GLI2012 versus Viljanen1982 *p*<0.001. LLN=lower limit of normal. The Viljanen1982 reference values provide predictions for adults 18–65 years of age (12). Males, *n*=597; females, *n*=731; in the age category 21–65 years: males *n*=512, females *n*=655; 2.5%=2.5th percentile; 5.0%=5th percentile. For the Kainu2015 and GLI2012 reference values the LLN for 2.5th percentile is z-score <−2 and the LLN for 5th percentile is z-score <−1.645. For the Viljanen1982 reference values the 2.5th percentile is defined as FEV1/FVC<88% predicted, FVC<80% predicted, and FEV1<80% predicted.

**Fig. 2:**
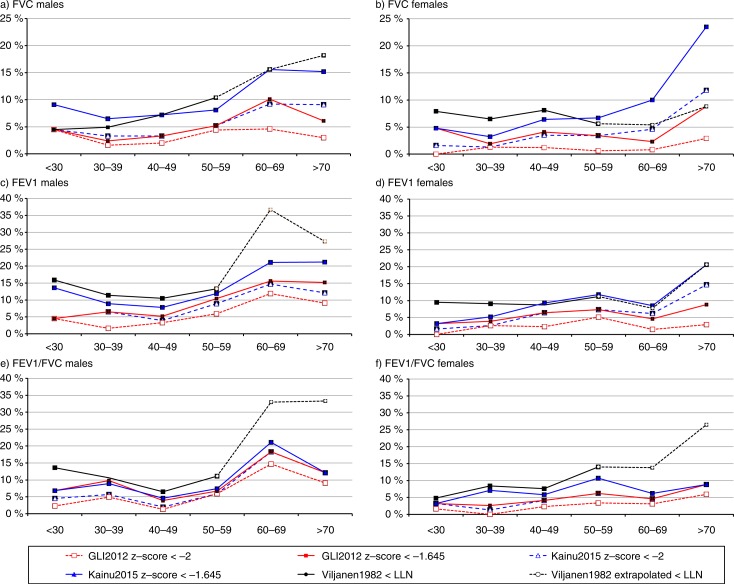
Prevalence of reduced FVC (a and b), FEV1 (c and d), and FEV1/FVC ratio (e and f) in different age categories stratified by sex using the GLI2012 (4), Kainu2015 (14), and Viljanen1982 (12) reference values. For the GLI2012 and Kainu2015 reference values, the 2.5th percentile limit of z-score <−2 and 5th percentile limit of z-score <−1.645 are shown. For the Viljanen1982 reference values, the lower limit of normal (LLN) was defined as 2.5th percentile. The Viljanen1982 reference equations apply for adults 18–65 years of age, but are currently used in clinical practice also for the elderly. Extrapolated values are indicated with the black dashed line.

The prevalence of obstruction, decreased ventilatory capacity, and possible restrictive pattern using the evaluated reference equations in age groups are illustrated in Fig. 2. The Viljanen1982 values are valid only up to 65 years and thus prevalences for each reference standard are also reported separately for the age group of 21–65 years in Table 2, and the extrapolated range is shown as dotted lines in Fig. 2.

In the complete study sample, 4.0, 5.6, and 13.0% of subjects were classified as obstructed in baseline spirometry with the GLI2012, Kainu2015, and Viljanen1982 reference values, respectively. Decreased ventilatory capacity was found in 4.0% of subjects with GLI2012, 6.9% with Kainu2015, and 13.3% with the Viljanen1982 reference values. Significantly more subjects (7.9%) were classified as having a possible restrictive pattern when using the Viljanen1982 reference values compared to 4.2% with the Kainu2015 and 2.0% with the GLI2012 values. In contrast, 0.8, 1.6, and 2.5% of subjects had only reduced FEV1 without reduced FEV1/FVC or FVC with the GLI2012, Kainu2015, and Viljanen1982 reference values, respectively.

In all evaluated reference equations, the prevalence of both obstruction and possible restrictive pattern of spirometry was more prevalent in males. With the Viljanen1982 reference values, the prevalence of possible spirometric restrictive pattern showed a significantly increasing trend with age in males. However, young females below 30 years of age also had a prevalence of up to 7.9%, compared to none (0.0%) with GLI2012 and 1.9% with the Kainu2015 values (*p*<0.001) (Fig. 2).

**Fig. 3:**
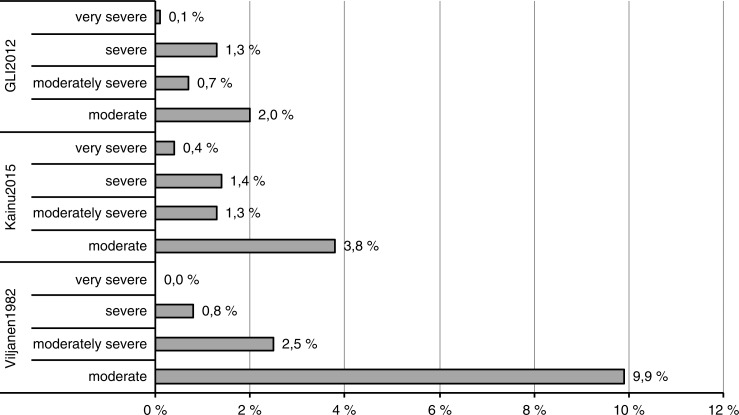
Prevalence of degrees of reduction in FEV1 in the general population using the GLI2012 (4), Kainu2015 (14), and Viljanen1982 (12) reference values. Reductions in the GLI2012 and Kainu2015 reference values graded according to Quanjer et al. (23). Reductions in the Viljanen reference values graded according to Halttunen and Sovijärvi (22).

The grading of reduced lung function with FEV1 by using z-scores and the modified grading for interpretation of the Viljanen1982 reference value (2) are shown in Fig. 3. The GLI2012 values produced systematically lower lung volume values and thus also lower rates of reduced lung volumes. The prevalence of moderate reduction in lung function was more common in the Viljanen1982 reference values: up to 12.4% of males had moderately reduced FEV1, compared to 4.5% with the Kainu2015 values and 2.5% with the GLI2012 values. For grading of the levels of reduced lung function with FEV1, the kappa statistic of agreement was 0.513 for Viljanen1982 and 0.587 for GLI2012, compared to the Kainu2015 predictions.

Overall the evaluated reference equations provided very good to fair agreement between categorisations in lung function abnormalities, with the kappa values showing the best agreement between the Kainu2015 and GLI2012 reference values at 0.86 (5th percentile limit) for FEV1/FVC and 0.73 for FEV1<LLN. On all evaluated models shown in Table 3, males had better levels of agreement between the different models, with the exception of reduced FEV1, where females had a better level of agreement between the Kainu2015 and Viljanen1982 reference values of the kappa value: 0.72 in females, compared to 0.59 in males.

**Table 3:** Levels of agreement for obstruction (FEV1/FVC<LLN), possible restrictive pattern (FVC<LLN), and decreased ventilatory capacity (FEV1<LLN) comparing each of the three reference equations with each other

		Kappa statistic for agreement								
		
		FEV1/FVC<LLN			FVC<LLN			FEV1<LLN		
				
	LLN (%)	Male	Female	All	Male	Female	All	Male	Female	All
Kainu2015 (14) vs. GLI2012 (4)	5	0.94	0.75	0.86	0.68	0.63	0.66	0.82	0.76	0.79
Kainu2015 (14) vs. GLI2012 (4)	2.5	0.89	0.70	0.82	0.75	0.43	0.62	0.80	0.63	0.73
Viljanen1982 (12) vs. GLI2012 (4)	2.5	0.53	0.32	0.44	0.48	0.24	0.38	0.43	0.41	0.43
Kainu2015 (14) vs. Viljanen1982 (12)	2.5	0.61	0.52	0.57	0.69	0.58	0.64	0.59	0.72	0.65

## Discussion

Both the international GLI2012 reference values and the new Finnish reference values (4, 14) produce lower prevalences of both airflow limitation and possible restrictive pattern and less milder degrees of decreased ventilatory capacity than the previously used Finnish national reference values proposed by Viljanen. There is no golden standard for the true prevalence of reduced lung function in the general population, which makes evaluation of the representativeness of reference values difficult. Traditionally, reference values are evaluated in samples of healthy non-smoking subjects, but with this approach various selection biases can significantly affect results. Amongst healthy non-smoking subjects, reference values should produce estimates equivalent to the LLN chosen and, in general, the prevalence would be expected to be higher in the general population.

Using the 2.5th percentile, the LLN definition of the Viljanen1982 values, the prevalence of airflow limitation of 13.0%, possible spirometric restrictive pattern of 7.9%, and decreased ventilatory capacity of 13.3% would seem to be very high for the general population. The GLI2012 values produced lower estimates of lung volumes than the Kainu2015 or Viljanen1982 values, with a greater difference especially in the older age categories. Using the GLI2012 equations, 4.0% of the people in the general Finnish population showed airway obstruction, 2.0% possible restrictive pattern, and 4.0% decreased ventilatory capacity, prevalences that appear to be too low given the definition of the 2.5th percentile limit for the LLN. In particular, the GLI2012 predicted values for females produced only 2.3% prevalence for obstruction and 1.0% for possible restrictive pattern. The GLI2012 equations failed to find 32% of cases of obstruction and 55% of cases of possible spirometric restrictive pattern compared to the new Finnish values by Kainu. Thus, the findings from our random population sample are in line with the findings of the study analysing the reference values in healthy non-smokers, which found that the GLI2012 equations underestimated lung volumes in healthy Finnish adults by approximately 5% (14).

The new national reference values by Kainu et al. reduced the estimates of both obstructive and restrictive findings by 50% compared to the currently used Viljanen1982 values. Prevalences of 5.6% for airway obstruction, 4.2% for possible restrictive pattern, and 6.9% for decreased ventilatory capacity would seem to be in line with the expected level of prevalence in the general population. Extrapolation of the equations by Viljanen to ages over 65 years, beyond the actual measurements of the original data set, increased the rate of misclassifications. Limiting the comparison of the Viljanen1982 values to the age category 21–65 years, as shown in Table 2, still results in approximately 100% larger prevalence estimates of both obstruction and possible restrictive pattern compared to the comparable 2.5th percentile LLN of the Kainu2015 values. The prevalence of borderline obstruction and possible spirometric restrictive pattern by the Viljanen1982 equations was higher also in younger age categories, particularly in males as shown in Fig. 2.

When interpreting the individual results in clinic, accurate estimates of normal values and LLN should be used. In Finland, the Viljanen1982 values used the 2.5th percentile to define the LLN. The change to using the 5th percentile LLN as recommended both by the ATS/ERS Task Force, in the previous ATS 1994 standard, and by the GLI2012 document for symptomatic subjects (4, 20, 21) will potentially result in greater numbers of healthy individuals being labelled as having pathological values. However, the present study demonstrates that the new national reference values with 5th percentile LLN produce still slightly lower levels of abnormal findings as currently found with the Viljanen1982 values (Table 2). The difference with GLI2012 results from older females in whom the GLI2012 values underestimate lung volumes and to a lesser degree also obstruction. It have been suggested that this is caused by secular trends in height and the Cormic index, as recently demonstrated in Japan (17). In addition, among healthy non-smokers in Northern Sweden and Brazil, the GLI2012 predictions have been found to underestimate lung volumes in a similarly age-dependent way (8, 15).

In the study of GLI2012 reference values, FEV1/FVC was found to be the least dependent on ethnicity (4). Our findings are in line with these findings, with the prevalence of reduced FEV1/FVC being closer in the new Finnish and the GLI2012 reference values, in both evaluated LLN criteria, than the lung volumes. However, using the GLI2012 reference, the prevalence of obstruction was lower than expected among females, which contrasts with the findings in Sweden, where the GLI2012 reference values were found to overestimate the prevalence of obstruction in healthy, female non-smokers (8).

Because the predicted FEV1 with the GLI2012 and Kainu2015 equations were smaller than those predicted by the Viljanen1982 equations in the 12–74-year-old population samples studied, the prevalence of mild to moderately decreased ventilatory capacity was systematically highest with the Viljanen1982 equations. Given the 2.5th percentile LLN and the corresponding categorisation of values below this threshold as moderately reduced, the greatest difference was seen in the moderately decreased ventilatory capacity, which was found in 9.9% with the Viljanen1982 values, in 3.8% with the Kainu2015 values, and in 2.0% with the GLI2012 values. This difference between reference standards disappeared in the severely decreased ventilatory capacity; thus the greatest differences are in the borderline category. The large differences in the milder categories have a large impact on clinical assessment, since cases previously classified as mild reductions with the Viljanen values will be classified as normal when using the new Kainu values; large differences in the prevalence estimates of common lung conditions will result. Our findings are in line with those from Sluga et al. reporting milder gradings of severity of obstruction in primary care when changing from the ECSC or Swanney et al. equations to the GLI2012 reference values in the Netherlands (7).

This study represents a random general population from two diverse locations in Finland: urban Helsinki and the rural Kemi region. A non-responder study was conducted in Kemi, which found the responders to be representative of the general population despite younger male smokers being less likely to attend (26). The study participants were found to correspond well to the age and sex distribution of the population at the time and the smoking prevalences to other studies from the same time period (27). The study measurements were undertaken at a research laboratory with quality control protocols tighter than usually available for epidemiological studies. Anthropometric height and weight data were measured by the research nurses, but unfortunately no sitting height or Cormic index was recorded, which limits analyses regarding the explanatory role of relative height. From both centres, Helsinki and Kemi, 403 of the 1,328 studied subjects were found to be healthy and non-smoking and were included in the dataset, from which new reference value models were derived, which can be seen as a limitation of this study. The total sample size in the reference values study was 1,000 adults, also including two other centres, Kuopio and Tampere (14). Evaluation of reference values in selected healthy non-smoking populations is inherently affected by the chosen selection criteria – the level of exposures, symptoms, or other diseases permitted. In order to assess the applicability of reference values in the general population, a random population sample is most representative aiming for the least selection biases. This study aimed at evaluating the practical implications of change in the reference values used and the level of LLN from the 2.5th percentile to the 5th percentile at the population level. Further study on non-related population samples is needed to verify our findings.

The age- and sex-related difference in lung volumes found here warrants further study at the population level into explanatory factors that need to be taken into consideration when interpreting lung function at the individual level. The Cormic index has rarely been included in measurements of lung function but to validate the links from other data suggesting secular and mostly non-linear trends between age and lung volumes, new research into this and possible other explanatory factors is needed to better understand variations in what should be considered normal at the population and individual levels.

There are great benefits in developing joint reference standards for lung function testing. However, recent research like ours has found the GLI2012 to poorly represent evaluated populations. This is not surprising given the wide range of populations joined for combined reference values. For clinical practice, national reference values are needed until additional factors, like the Cormic index, are found that could better explain the variation found between populations. In Finland, national reference values have been used uniformly in all laboratories with national recommendations published regularly, which has limited interpretative differences within the country. Since new Finnish reference values have been developed with mathematical modelling similar to the GLI2012 model, we find that the grading of reduced lung function proposed by Quanjer et al. can be used with both reference models (23). New Finnish reference values are suggested for use for adult native Finns, with GLI2012 to be used for all other ethnicities. Use of the ECSC reference values should be discontinued. Further research into incorporating different populations into separate GLI models is needed.

## Conclusions

In this study, we showed lower prevalences of a possible restrictive pattern (reduced FVC), airflow limitation (reduced FEV1/FVC ratio), and decreased ventilatory capacity (reduced FEV1) in two random Finnish general population samples when using the international GLI2012 values compared to the new Kainu2015 values and the old Viljanen1982 national values. The application of the GLI2012 reference values for native Finns resulted in lower-than-expected prevalence estimates, especially in the category of possible restrictive patterns. The adoption of the new Kainu2015 reference values for native Finns instead of the old Viljanen1982 values will markedly reduce the prevalence of obstruction, possible restrictive pattern, and decreased ventilatory capacity in the general population, by up to 50%, mostly from the borderline and milder categories. The study compared fitting of the three reference equations into general population samples consisting of native 21–74-year-old Finns. The results favour implementation of the Kainu2015 values, both for clinical use to avoid false positive and negative findings of lung function disturbances and for epidemiological studies to achieve more reliable figures of prevalences of airways obstruction and restrictive impairment.

## Supplementary Material

Prevalence of abnormal findings when adopting new national and international Global Lung Function Initiative reference values for spirometry in the Finnish general population
