# Supplementary material for: Prevalence of abnormal findings when adopting new national and international Global Lung Function Initiative reference values for spirometry in the Finnish general population
Source: Eur Clin Respir J. 2016 Sep 6;3:10.3402/ecrj.v3.30658. doi: 10.3402/ecrj.v3.30658 (PMC5015641; doi:10.3402/ecrj.v3.30658)
Supplement: Prevalence of abnormal findings when adopting new national and international Global Lung Function Initiative reference values for spirometry in the Finnish general population [file ECRJ-3-30658-s001.docx]

**Annex 1.**

**Details of measurement protocol of the study**

Anthropometric measurements were conducted in light clothing without shoes. Height was measured standing with heels together, heels and back lined with the wall, and head held in Frankfurt plane. Height was measured with a 200cm wall mounted height meter ruler stadiometer (KaWe Person-Check, Kirchner&Wilhelm GmbH, Asperg, Germany) to 0.1cm accuracy. In subjects unable to stand, armspan was used as a proxy. Weight was measured using a digital scale (Seca 878, seca, Birmingham, UK) to 0.1kg accuracy. Both height and weight were measured once before spirometry.

A Sensor Medics VMax 22D flow-volume spirometer (Vmax 22D, Sencor Medics, Yorba Linda, CA, USA) was used. Spirometry equipment was quite new at the time of the study and did not need any servicing during the study. The equipment measures ambient temperature and barometric pressure, which were calibrated initially by manufacturer and thereafter during maintenance. The BTPS conversion was done according to ATS 1994 standard (American Thoracic Society, 1995). Calibration of the spirometer was checked with a 3-L calibration syringe (Sensor Medics) once a day and whenever the spirometer software requested calibration. Calibration syringe was stored in the same air conditioned controlled laboratory environment as the spirometer equipment, and operated according to manufacturer’s instructions to ensure accuracy. Manufacturer recommended filters of MicroGard bacterial/viral filters (model number 769204) were used. This model has resistance of 0.7 cmH2O/l/sec (tested at 12 litres/sec) both in expiration and inspiration. Spirometry was completed in seated position using a nose clip. Spirometry maneuvers were completed using ATS 1994 criteria (American Thoracic Society, 1995), but the repeatability criteria were determined according to the 1993 ERS standard (Quanjer PH et al., 1993). Repeatability criteria used were: the two largest FEV1 and FVC values were required to be within 5% of the respective volume or within 100ml, whichever was greater, and the two largest peak expiratory flow (PEF) values were required to be within 10% of each other. In addition, each of the curves used for analysis should fulfill the quality criteria concerning extrapolated volume (Quanjer et al., 1993). At least three technically acceptable measurements were recorded with a maximum of eight efforts. Three experienced study nurses performed the measurements, two in Helsinki and one in Kemi.

**References**

American Thoracic Society. Standardization of spirometry. 1994 Update. Am J Respir Crit Care Med 1995; 152: 1107-1136.

Quanjer PH, Tammeling GJ, Coters JE, Pedersen OF, Peslin R, Yernault J-C. Lung Volumes and Forced Ventilatory Flows. Report Working Party. Official Statement of the European Respiratory Society. Eur Respir J 1993; 6, Suppl. 16: 5-40.
